# TrypanocidalActivity of Natural Sesquiterpenoids Involves Mitochondrial Dysfunction, ROS Production and Autophagic Phenotype in *Trypanosoma cruzi*

**DOI:** 10.3390/molecules23112800

**Published:** 2018-10-28

**Authors:** Ana Cristina Souza Bombaça, Daniela Von Dossow, Juliana Magalhães Chaves Barbosa, Cristian Paz, Viviana Burgos, Rubem Figueiredo Sadok Menna-Barreto

**Affiliations:** 1 Laboratório de Biologia Celular, Instituto Oswaldo Cruz, Fundação Oswaldo Cruz, Rio de Janeiro 21040-360, Brazil; anabombaca@gmail.com (A.C.S.B.); julianabmunirio@gmail.com (J.M.C.B.); 2 Departamento de Ciencias Químicas y Recursos Naturales, Universidad de La Frontera, Temuco 4811230, Chile; d.vondossow01@ufromail.cl (D.V.D); viviana.burgos@ufrontera.cl (V.B.)

**Keywords:** *Trypanosoma cruzi*, chagas disease, chemotherapy, sesquiterpenoids, mitochondria, autophagy

## Abstract

Chagas disease is a neglected tropical disease that is caused by the protozoan *Trypanosoma cruzi* and represents a serious health problem, especially in Latin America. The clinical treatment of Chagas disease is based on two nitroderivatives that present severe side effects and important limitations. In folk medicine, natural products, including sesquiterpenoids, have been employed for the treatment of different parasitic diseases. In this study, the trypanocidal activity of compounds isolated from the Chilean plants *Drimys winteri*, *Podanthus mitiqui* and *Maytenus boaria* on three *T. cruzi* evolutive forms (epimastigote, trypomastigote and amastigote) was evaluated. Total extracts and seven isolated sesquiterpenoids were assayed on trypomastigotes and epimastigotes. Polygodial (Pgd) from *D. winteri*, total extract from *P. mitiqui* (PmTE) and the germacrane erioflorin (Efr) from *P. mitiqui* were the most bioactive substances. Pgd, Efr and PmTE also presented strong effects on intracellular amastigotes and low host toxicity. Many ultrastructural effects of these substances, including reservosome disruption, cytosolic vacuolization, autophagic phenotype and mitochondrial swelling (in the case of Pgd), were observed. Flow cytometric analysis demonstrated a reduction in mitochondrial membrane potential in treated epimastigotes and an increase in ROS production and high plasma membrane permeability after treatment with Pgd. The promising trypanocidal activity of these natural sesquiterpenoids may be a good starting point for the development of alternative treatmentsforChagas disease.

## 1. Introduction

Chagas disease is a parasitic infection caused by the haemoflagellate protozoan *Trypanosoma cruzi* that poses a serious health problem in Latin America [1]. This illness is classified as one of the most neglected tropical diseases, causing significant mortality and morbidity, especially among low-income populations [2]. However, Chagas disease is not restricted to Latin American countries. In recent decades, the migratory flux of infected people from endemic areas to Europe or the United States, among other areas, has allowed the occurrence of new cases in non-endemic countries related to blood donation and/or vertical transmission [3,4]. This illness presents two clinical phases. In the acute stage, a high patent parasitemia is detected in the short period after the infection, without remarkable and specific symptoms [5]. Following the infection course, the chronic phase begins;this phase is defined by the absence of symptoms and positive serology [6]. After decades in the indeterminate chronic phase, some of the patients (30–40%) will progress to the symptomatic stage, where cardiomyopathy is the most frequent clinical manifestation [5]. In regards to chemotherapy, only two nitroderivatives—benznidazole and nifurtimox—are clinically available for the treatment of Chagas disease. Both present high toxicity and undesirable side effects as well as limited efficacy in the chronic phase, justifying the continuous search for novel drugs [7,8].

The *T. cruzi* life cycle is characterized by three parasite stages: two clinically relevant forms (infective trypomastigotes and intracellular amastigotes) and the insect form (epimastigotes). Inside triatomine insects, epimastigotes proliferate in the midgut. The protozoa then migrate to the insect rectum, and differentiation into the metacyclic trypomastigotes that may infect the mammalian host occurs. Once inside a mammalian cell, the parasite undergoes another differentiation, now to the amastigote form, which is responsible for *T. cruzi* replication in vertebrates. After some mitotic divisions, amastigotes differentiate into trypomastigotes that then reach the bloodstream, disseminating the infection. In the search for alternative drugs, ultrastructural analysis of novel trypanocidal drugs is fundamental for the identification of specific targets such as molecules, organelles or biochemical events in this protozoan parasite [9,10].

Trypanosomatids, including *T. cruzi,* contain a unique mitochondrion that is associatedwith the parasite’s rudimentary antioxidant defences, making this organelle and oxidative molecules interesting targetsfor drug intervention [11,12]. The mitochondrial electron transport chain is a crucial checkpoint for redox processes, representing one of the main sources of reactive oxygen species (ROS) in the parasite. Another biological pathway that has been associated with the mechanisms of action of anti-trypanosome drugs is autophagy [13,14]. The autophagy pathway is a pivotal degradation process in the recycling of cellular structures, which is also important in trypanosomatids [15]. The loss of autophagic balance could represent a critical point for the development of chemotherapeutic alternatives.

In recent decades, the use of natural products for the treatment of parasitic diseases has been considered as an alternative approach [16]. Looking for new natural compounds that act on *T. cruzi*, we focused on natural sesquiterpenoids from Chilean flora, particularly drimane sesquiterpenoids from *Drimys winteri*, germacrane sesquiterpenoids from *Podanthus mitiqui* and dihydro-β-agarofuran sesquiterpenoids from *Maytenus boaria*, which have shown a broad range of activities.

*D. winteri* J.R. (Winteraceae), usually called Canelo, is a native tree of south-central Chile and Argentina. The native people of Chile, called *Mapuche,* consider this tree sacred; a symbol of benevolence, peace and justice. It is present at all social and religious meetings, called “guillatún” and “machitún”, where the healer, or “machi”, uses Canelo leaves or sap as medicine. Among the three products of focus here, drimane sesquiterpenoids stand out as secondary metabolites withpotent fungicidal, anti-feeding and insecticidal activities, and drimane sesquiterpene lactones have demonstrated activity on bacterial quorum sensing [17,18]. *P. Mitiqui* (Lindl) (Asteraceae, Compositae) is another endemic plant of the Central Zone of Chile. This plant produces sesquiterpene lactones with a germacrane framework, such as ovatifolin and erioflorin [19], which show cytotoxic and antiprotozoal activities [20,21]. In addition, previous reports of natural compounds purified from *M. boaria* Mol. (Celastraceae) showed highly oxygenated sesquiterpenes with a dihydro-*β*-agarofuran skeleton, which have been shown to display activity in reversing the drug resistance of human leukaemia [22]. In the present study, we extended our investigation to the trypanocidal activity of total extracts and their respective isolated sesquiterpenoids, evaluating the mechanisms of action of the most active compounds with electron microscopy and flow cytometry approaches.

## 2. Results

**Figure 1:**
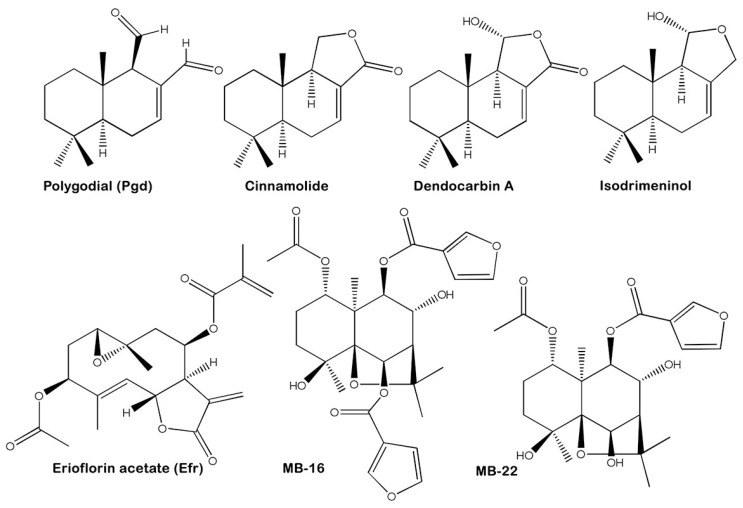
Molecular structures of sesquiterpenoids. Drimane sesquiterpenoids, including polygodial (Pgd), cinnamolide, dendocarbin A and isodrimeninol were purified from the bark of *D. winteri*. From *P. mitiqui,* the germacrane sesquiterpenoid erioflorin acetate (Efr) was isolated. The β-dihydroagarofuran compounds MB-16 and MB-22 were purified from *M. boaria.*

First, the drimane sesquiterpenoids polygodial (Pgd, [(1*R*,4a*S*,8a*S*)-1,4,4a,5,6,7,8,8a-octahydro-5,5,8a-trimethylnaphthalene-1,2-dicarbaldehyde]), cinnamolide [(5a*S*,9a*S*,9b*R*)-1,5a,6,7,8,9,9a,9b-octahydro-6,6,9a-trimethylnaphtho[2,1-*c*]furan-3(5*H*)-one], dendocarbin A [(1*R*,5a*S*,9a*S*,9b*R*)-1,5a,6,7,8,9,9a,9b-octahydro-1-hydroxy-6,6,9a-trimethyl-naphtho[2,1-c]furan-3(5*H*)-one], and isodrimeninol [(1*R*,5a*S*,9a*S*,9b*R*)-6,6,9a-trimethyl-1,3,5,5a,6,7,8,9,9a,9b-decahydronaphtho[1,2-c]furan-1-ol] (Figure 1) were isolated from ethyl acetate (EtOAc) extracts of the bark from the Canelo tree, *D. winteri.* These substances were chemically characterized using X-ray analysis as a *trans*-decalin moiety with a Δ^7(8)^ double bond, in a twisted chair conformation at the second ring. The germacrane sesquiterpene erioflorin acetate (Efr, [(1a*R*,3*S*,5a*R*,8a*R*,9*R*,10a*S*,*E*)-3-acetoxy-4,10a-dimethyl-8-methylene-7-oxo-1a,2,3,5a,7,8,8a,9,10,10a-decahydrooxireno[2’,3’:5,6]cyclodeca[1,2-*b*]-furan-9-yl methacrylate]) (Figure 1) was isolated from aerial parts of *P. mitiqui*. Its structure was confirmed by X-ray analysis as a central ten-carbon ring with two heterocycles (an epoxide between C1 and C10 and a butyrolactone between C6 and C7) and a five-carbon unsaturated ester side-chain attached to C8. The dihydro-β-agarofuran sesquiterpenoids MB-16 [(1*S*,4*S*,5*S*,6*R*,7*R*,8*R*,9*R*,10*S*)-6-acetoxy-4,9-dihydroxy-2,2,5a,9-tetramethyloctahydro-2*H*-3,9a-methanobenzo[b]oxepine-5,10-diyl bis-(furan-3-carboxylate)] and MB-22 [(1*S*,4*S*,5*S*,6*R*,7*R*,9*S*,10*S*)-6-acetoxy-9-hydroxy-2,2,5a,9-tetramethyl-octahydro-2*H*-3,9a-methanobenzo-[b]oxepine-5,10-diyl bis-(furan-3-carboxylate)] (Figure 1) were isolated from seeds of *M. boaria* macerated with EtOAc. These compounds have a three-cycle structure formed by a central decalin system bound to an ether at C5 and C7, where C5 is esterified with an acetate and C9 with a furoate. However, only MB-16 contains a second furoate at C6, resulting in steric hindrance on the α side of the molecule. MB-22 was previously chemically characterized [23]. The ^1^H nuclear magnetic resonance (NMR) and ^13^C-NMR data also confirm all chemical structures studied in this work for the active compounds Pgd and Efr (Figures S1 and S2 in Supplementary File).

In the present study, the trypanocidal efficacy of total extracts of *D. winteri*, *P. mitiqui* and *M. boaria* and the isolated sesquiterpenoids on *T. cruzi* bloodstream trypomastigotes and epimastigotes was assessed (Table 1). Against bloodstream forms, Pgd was the most active substance derived from *D. winteri* (IC_50_/24 h = 11.5 ± 2.1 µg/mL). *P. mitiqui* total extract (PmTE) and the respective purified Efr presented high anti-trypomastigote effects, presenting IC_50_/24 h values of 5.6 ± 0.6 and 6.1 ± 1.6 µg/mL. On epimastigotes, Pgd, PmTE and Efr were also the most active substances, with IC_50_/24 h values of 84.4 ± 9.4, 40.8 ± 5.3, 55.4 ± 5.4 µg/mL, respectively. Regarding *M. boaria*, the total extract and the isolated sesquiterpenoids presented no trypanocidal activity on infective and insect forms of the parasite. Pgd, PmTE and Efr were also strongly active on intracellular amastigotes, with IC_50_/24 h values of approximately 1 µg/mL. PrestoBlue assays demonstrated host toxicity of these three substances in concentrations much higher than the IC_50_/24 h for intracellular forms (LC_50_/24 h in the range of 10–25 µg/mL), expressed in selectivity index (SI) values of 10.3, 21.5 and 29.5-fold for Pgd, PmTE and Efr, respectively (Table 1).

**Figure 2:**
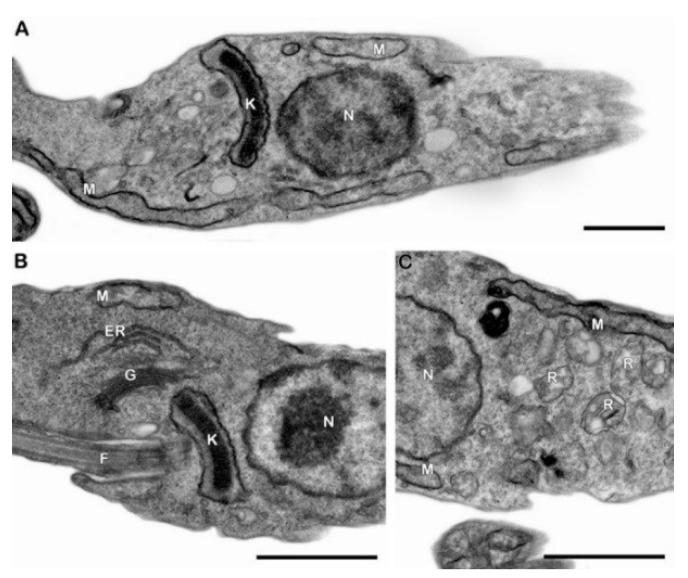
TEM analysis of untreated *T. cruzi* epimastigotes. (**A**–**C**) Control parasites showing normal ultrastructural aspects of nucleus (N), mitochondrion (M), kinetoplast (K), Golgi (G), endoplasmic reticulum (ER), flagellum (F) and reservosomes (R). Bars = 1 µm.

To assess the mechanisms of action of Pgd, PmTE and Efr, transmission electron microscopy (TEM) and flow cytometry approaches were employed. First, the ultrastructural analysis of non-treated epimastigotes showed typical morphology, such as an elongated unique mitochondrion and spherical, electron-dense reservosomes, with classical lipid inclusions inside (Figure 2). 

**Figure 3:**
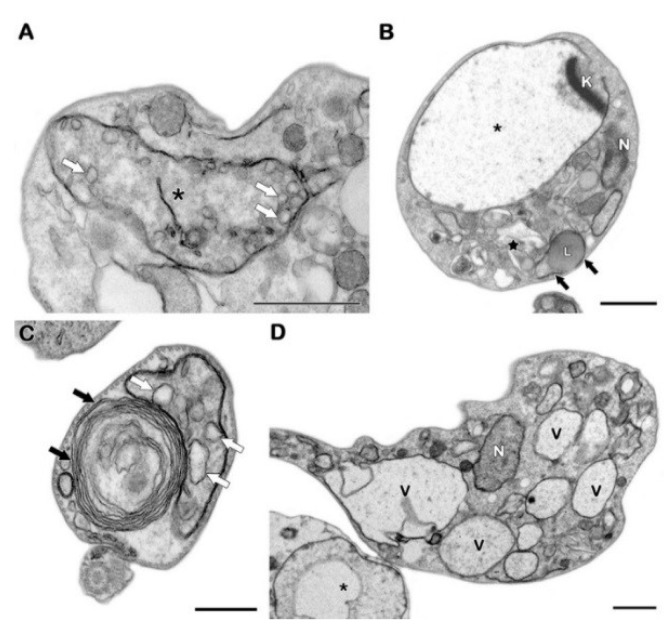
TEM analysis of *T. cruzi* epimastigotes treated with 42 µg/mL drimane sesquiterpenoid Pgd. (**A**–**D**) The treatment induced remarkable mitochondrial swelling (black asterisks), with dilation of the mitochondrial cristae (white arrows) and reservosome disorganization (black star). Endoplasmic reticulum profiles (black arrows) can be observed in treated parasites surrounding cytoplasmic portions and/or other cellular structures, including lipid droplets (L). Pgd also led to intense cytosolic vacuolization (V). N: nucleus, K: kinetoplast. Bars in A, B and D = 1 µm; bar in C = 0.5 µm.

**Figure 4:**
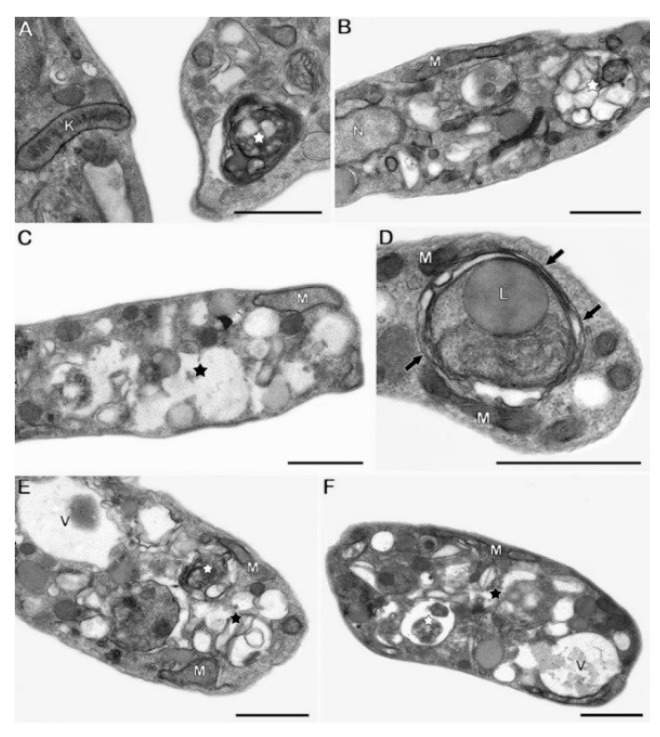
TEM analysis of *T. cruzi* epimastigotes treated with Efr. The treatment with (**A**–**C**) 25 and (**D**–**F**) 50 µg/mL of this germacrane sesquiterpenoid induced the appearance of numerous autophagosomes (white stars) and the formation of endoplasmic reticulum profiles (black arrows) in close contact with subcellular structures such as lipid droplets (L). Parasites treated with Efr also showed reservosome disorganization (black stars) and cytosolic vacuolization (V). N: nucleus, M: mitochondrion, K: kinetoplast. Bars = 1 µm.

Treatment with 42 µg/mL of Pgd for 24 h led to severe mitochondrial swelling, as demonstrated by cristae dilatation and intense cytosolic vacuolization. The reservosomal morphology was also completely altered by the compound, and the presence of prominent endoplasmic reticulum profiles surrounding different cellular structures was detected in treated parasites (Figure 3). Similar morphological phenotypes were also observed after incubation with 85 µg/mL of this sesquiterpenoid (Figure S3 in Supplementary File 1). On the other hand, Efr (25–50 µg/mL) induced a significant increase in the number of autophagosomes, evidenced by the detection of endoplasmic reticulum profiles surrounding parasite structures, such as lipid droplets. As observed after treatment with Pgd, epimastigotes treated with Efr also presented strong cytosolic vacuolization and reservosome disruption (Figure 4). The same ultrastructural effects detected in Efr-treated parasites (autophagic features, reservosome alterations and vacuolization) were also found after treatment with the corresponding total extract (20–40 µg/mL PmTE) (Figure S4 in Supplementary File 1).

**Figure 5:**
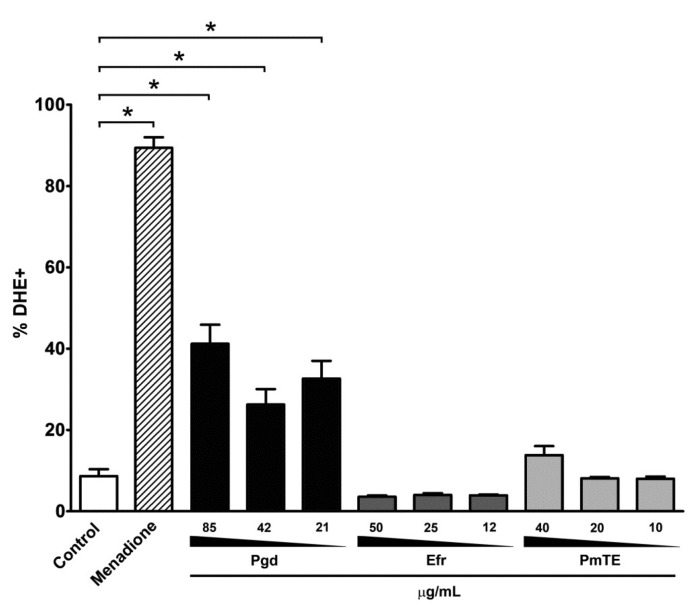
Flow cytometry analysis of ROS production in *T. cruzi* epimastigotes by DHE labelling. Pgd led to an increase in the percentage of parasites generating ROS. As a positive control, 10 µM menadione was employed. The graphs present the mean and standard deviation of least three independent experiments. * Asterisks represent the significant difference in relation to the untreated control group (*p* ≤ 0.02) by Mann-Whitney test.

Rhodamine 123 (Rh123) analyses revealed a reduction of marker fluorescence after treatment with Pgd, Efr and PmTE, expressed in index of variation (IV). ΔΨm was dose dependent in treated parasites, decreasing the fluorescence in 83% (Pgd), 90% (Efr) and 94% (PmTE) at IC_50_/24 h concentrations (Table 2). The use of dihydroethidium (DHE) demonstrated an important dose-dependent increase in ROS production in Pgd-treated parasites, enhancing up to 4.8-fold the percentage of labelled epimastigotes in comparison to control protozoa, up to 40% at the dose of 85 µg/mL (Figure 5). 

**Figure 6:**
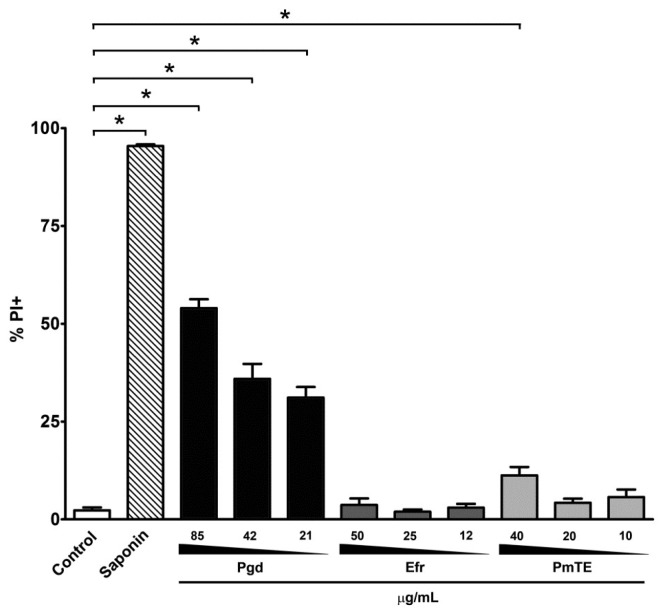
Flow cytometry analysis of plasma membrane integrity in *T. cruzi* epimastigotes by PI labelling. Treatment with Pgd induced permeabilization of the parasite plasma membrane. As a positive control, epimastigotes were incubated with 0.1% saponin. The graphs present the mean and standard deviation of four independent experiments. *Asterisks indicatesignificant difference in relation to the untreated control group (*p* ≤ 0.03) by Mann-Whitney test.

Interestingly, when examining the intensity of DHE labelling, the treatments with two of the sesquiterpenoids, Pgd and Efr, and the total extract PmTE, led to a large dose-dependent increase in the marker fluorescence, in the range of 5.9–6.2 (21–85 µg/mL), 3.1–4.7 (12–50 µg/mL) and 3.1–3.6-fold (10–40 µg/mL) (Table 3). The propidium iodide (PI) assays showed a dose-dependent permeabilization of epimastigotes treated with Pgd, reaching 54% of parasites PI+ at a dose of 85 µg/mL. However, treatment with PmTE and Efr at concentrations up to IC_50_/24 h did not result in an important loss of plasma membrane integrity in the protozoa (Figure 6).

## 3. Discussion

The biological activity of natural sesquiterpenoids has been extensively investigated, including in trypanosomatids [24,25,26,27]. Initially, we tested total extracts of three Chilean plants and isolated sesquiterpenoids on infective bloodstream trypomastigotes and proliferative epimastigotes. Among the assayed substances, Pgd, Efr (isolated compounds) and PmTE (total extract) were the most active on both parasite forms and were selected for the subsequent analysis, as we discuss below. Clearly, the clinically relevant forms of *T. cruzi* were more susceptible to these three substances than was the insect form. For trypomastigotes, IC_50_/24 h values oscillated from 5 to 12 µg/mL, while the range was 5- to 12-fold lower for amastigotes, suggesting that microbicidal activity of macrophages could potentiate the anti-*T. cruzi* effect. The host toxicity evaluation indicated concentrations 10- to 30-fold higher than the IC_50_ doses for intracellular forms, reinforcing the selectivity of these substances. In a previous study, Pgd was purified from *Drimys brasiliensis* and assayed on different forms of three *Leishmania* spp. and *T. cruzi* cultured-derived trypomastigotes [24]. The IC_50_ values for *Leishmania* promastigotes varied from 35 to 65 µg/mL, in the same range as described in this work for our three selected substances on the proliferative insect form (40–85 µg/mL). Evaluating the effect of Pgd on trypomastigotes, Corrêa et al. reported higher (approximately 6-fold) activity, but it was assayed on cultured-derived forms that were not exposed to the host immune system. Recent work from our group compared the proteomic profiles of bloodstream and cultured-derived trypomastigotes, showing more than 2000 proteins expressed exclusively in bloodstream forms [28].These data reinforce the differences between these forms, justifying the variation in susceptibility to Pgd. Indeed, considering these study recommendations, Pgd, Efr and PmTE are far from ideal to be considered promising alternatives for Chagas disease [29], but they could be an interesting starting point for the search for novel trypanocidal agents. For all three parasite stages, PmTE and Efr were discretely more active than Pgd, indicating a better trypanocidal effect of *P. mitiqui*. However, similar IC_50_/24 h values were obtained for this total extract and its purified sesquiterpenoid, suggesting that trypanocidal activity observed in the extract was probably derived from the presence of one or more active compounds, among them Efr, which is only in a 0.0052% yield (fresh plant). The potential synergistic/antagonistic effects of Efr and other components present in this total extract must be further analysed.

To assess the mechanism of action of Pgd, Efr and PmTE, treated epimastigotes were evaluated by transmission electron microscopy and flow cytometry techniques, never exceeding the IC_50_/24 h concentrations. The most common morphological phenotype detected after treatment with the three natural products was the exacerbation of the autophagic pathway, detected by the presence of a large number of autophagosomes as well as the appearance of well-developed endoplasmic reticulum profiles surrounding subcellular structures and organelles. Such bizarre membrane structures with a myelin-like aspect are very prominent. Autophagy is a well-regulated process that is crucial for remodelling cellular structures and maintaining homeostasis in all eukaryotic cells, and the loss of the autophagic balance leads to death [30]. In trypanosomatids, ATG orthologues have been described, and two ubiquitin-like conjugation systems have been detected [31,32]. In *T. cruzi*, the triggering of autophagic machinery has been reported, especially after treatment with different classes of drugs, including natural products such as naphthoquinones or geranylgeraniol [13,14,33,34]. In *T. cruzi*, the sesquiterpenelactones psilostachyin A and cynaropicrin induced anautophagic phenotype in bloodstream forms [35]. Our ultrastructural data reinforced the autophagic induction by sesquiterpenoids in this parasite and suggested the endoplasmic reticulum as the origin of the phagophore membrane [36]. Another interesting point to be mentioned is the importance of autophagic induction for differentiation processes in trypanosomatids, as demonstrated in *Leishmania* spp. [37]. The involvement of reservosomes in metacyclogenesis as well as their participation in autophagy has been postulated [32,38]. The severe reservosome membrane disruption induced by Pgd, Efr and PmTE also reinforced autophagy as part of the mode of action, suggesting that damage of this organelle leads to protease release into the cytosol, culminating in the death of the protozoa. Reservosome disorganization certainly compromises the differentiation into trypomastigotes, as was previously observed in naphthoimidazole-treated parasites [39], but further experiments must be performed in this direction.

Ultrastructural evidence also pointed to *T. cruzi* mitochondrion as a target severely affected by Pgd, as previously described in *Leishmania chagasi* promastigotes [24]. Mitochondrial swelling has been described as one of the most recurrent drug-induced alterations in *T. cruzi* [12,13,33,39,40]. Such organelles participate in ATP synthesis and in redox balance, being the main ROS source in the parasite. In trypanosomatids, the functional and structural peculiarities of the mitochondria and the ROS detoxification mechanisms signifythis organelle as a promising target for drug intervention [12]. The evaluation of ΔΨm revealed a strong depolarization of the mitochondrial membrane induced by Pgd, Efr and PmTE at sub lethal concentrations (25 to 60% of Rh123 fluorescence decrease at a quarter of IC_50_ doses), which confirmed the morphological phenotype, demonstrated the mitochondrial susceptibility and corroborated the hypothesis of this organelle as a primary target of these substances. Once the treatment strongly impaired ΔΨm, increased electron leakage from oxidative phosphorylation could be occurring, leading to ROS production. The analysis of ROS generation evidenced a remarkable increase in the fluorescence of the marker (3- to 6-fold higher than that of control parasites), suggesting an important increase in ROS levels in treated epimastigotes. On the other hand, the increase in the number of labelled parasites was only induced by Pgd, indicating that only this compound promotes an increase in both the percentage of epimastigotes producing ROS and the quantity of reactive species generated. Lipid peroxidation is a classical consequence of the increase in cellular ROS amounts, which can also lead to plasma membrane rupture [15]. Our results showed an increase in plasma membrane permeability and ROS production only after incubation with Pgd, which is indicative of necrosis. However, depending on the concentration, ROS can also trigger autophagy [41], affordinga plausible hypothesis for the mode of action of *P. mitiqui* extract and purified compound. Finally, the search for natural products, especially those with potential biological activities in folk medicine, could be an excellent alternative for the discovery of novel anti-*T. cruzi* drugs.

## 4. Materials and Methods

### 4.1. Purification and Chemical Analysis of Sesquiterpenoids

Drimane sesquiterpenoids were purified from *D. winteri* bark pieces collected in Temuco, IX Region of Chile, in February 2015. Bark (4.5 kg) was initially crushed and extracted by maceration with EtOAc (6 L) for 72h. The organic layer was evaporated *in vacuo*, giving a crude product (60 g) that was then further purified by column chromatography, giving eight primary fractions (F1–F8) with increasing polarity from hexane to EtOAc. A subsequent chromatographic purification of F3 with *n*-hexane/EtOAc (9:1 *v*/*v*) gave cinnamolide (320 mg, colourless crystals, 0.0071% yield), and the purification of F4 with *n*-hexane/EtOAc (8:2 *v*/*v*) gave Pgd (950 mg, yellow oil, 0.021% yield). Further purification of F5 by Sephadex Lh-20 gave isodrimeninol (500 mg, yellow oil, 0.011% yield), and the purification of F6 with *n*-hexane/EtOAc (1:1 *v*/*v*) gave dendocarbin A (60 mg, colourless crystals, 0.0013% yield).

Germacrane sesquiterpenoids were purified from aerial parts of *P. mitiqui* collected in Concepcion, VIII Region of Chile, in February 2015 (9.6 kg). Vegetal material was powdered and extracted by maceration with EtOAc for 3 days (8 L). The organic layer was evaporated *in vacuo* to give a crude product (250 g) that was further purified by column chromatography, giving 11 primaryfractions (F1–F11) with increasing polarity from *n*-hexane to EtOAc. F-7 (5 g) was further purified by column chromatography (silica gel 60/70-210 mesh, hexane/EtOAc 1:3 *v*/*v*) and gave Efr (500 mg, colourless crystals, 0.0052% yield).

Dihydro-β-agarofuran sesquiterpenoids were purified from *M. boaria* seeds (1.3 kg) collected in Temuco, IX Region of Chile, in September 2016. The seeds were crushed and extracted by maceration with EtOAc for 72h (4 L). The organic solvent was evaporated *in vacuo*, giving a crude extract (600 g, orange oil) that was further fractionated by silica gel column chromatography, giving nine primary fractions (F1–F9) by using increasing polarity from *n*-hexane/EtOAc. Fractions F1 to F4 contained carotenoids, unsaturated fatty acids and β-sitosterol, but no sesquiterpenes were detected. A subsequent purification of F7 by CC with *n*-hexane/EtOAc (2:1 *v*/*v*) gave BM 16 (55 mg, colourless crystals, 0.0092% yield), whereas the purification of F9 with *n*-hexane/EtOAc (1:1 *v*/*v*) gave BM 22 (180 mg, colourless crystals, 0.014% yield).

The retention factor in thin layer chromatography (TLC-Rf) of fractions and compounds were used in contrast with the Rf of pure standard, available in our labs, as a preliminary guide in all purification processes. Concentrates and pure compounds in solution were recrystallized by slow evaporation at 20 °C from mixtures of solvents, including hexane, EtOAc and methanol, to obtain colourless crystals suitable for single-crystal X-ray diffraction [23,42]. All compounds were confirmed by NMR in 1D and 2D. The ^1^H- and ^13^C-NMR spectra were recorded in CD_2_Cl_2_ (dendocarbin A, Efr, BM 16 and BM 22) or CDCl_3_ (Pgd, cinnamolide, isodrimeninol) solution in 5 mm tubes at room temperature on an Avance III spectrometer (BrukerBiospin GmbH, Rheinstetten, Germany) at 600.13 (^1^H) and 150.61 (^13^C) MHz, with the deuterium signal of the solvent as the lock and TMS (for ^1^H) or the solvent (for ^13^C) as internal standard. All spectra (^1^H, ^13^C, GS-H, H-COSY, edited HSQC, and GS-HMBC) were acquired and processed with the standard Bruker software. The ^1^H- and ^13^C-NMR spectra are provided in the Supplementary File.

For the biological assays, *D. winteri* total extract and four drimane sesquiterpenoids (Pgd, cinnamolide, dendocarbin A and isodrimeninol) were screened, as well as *P. mitiqui* total extract and the germacrane sesquiterpenoide Efr, *M. Boaria* total extract and the dihydro-β-agarofuran sesquiterpenoids in fractions F16 and F22. All stock solutions of the total extracts and sesquiterpenoids were prepared such thata final concentration of 0.5% dimethyl sulfoxide (DMSO) was not exceeded, to avoid damage caused by the solvent.

### 4.2. Animals and Parasites

Albino Swiss mice were employed for the purification of bloodstream trypomastigotes and for primary culture of macrophages. This work is in accordance with the guidelines of the Colégio Brasileiro de Experimentação Animal (COBEA) and was performed in biosafety conditions. All animal procedures were reviewed and approved by the Fiocruz Committee of Ethics in Animal Research (L-005/2017) according to resolution 196/96 of the National Health Council of Brazilian Ministry of Health.

All assays were performed with the *T. cruzi* Y strain. Bloodstream trypomastigotes were isolated from the blood of albino Swiss mice 7 d after intraperitoneal injection with 5 × 10^5^ parasites. Blood was collected by heart puncture, and the purified parasites were obtained by differential centrifugation (500× *g* for 30 min at 4 °C) [43]. Proliferative epimastigotes were maintained axenically at 28 °C in a liver infusion and tryptose (LIT) medium supplemented with 10% foetal bovine serum (FBS) (Cultilab, Campinas, Brazil). The medium was changed weekly, and epimastigotes were harvested during the exponential growth phase (5-day-old cultures).

### 4.3. Direct Effect on Bloodstream Trypomastigotes and Epimastigotes

The suspension of trypomastigotes or epimastigotes (10^7^ cells/mL) in RPMI or LIT medium, respectively, was added to the same volume of each of the 10 sesquiterpenoids, which had been previously prepared at twice the desired final concentrations and then incubated for 24 h at 37 °C (trypomastigotes) or 28 °C (epimastigotes) in 96-well microplates (Nunc Inc., Rochester, NY, USA). Parasite counts were performed in a Neubauer chamber. Compound activities correspond to the concentration that led to 50% lysis/proliferation inhibition of the parasites within 24 h (IC_50_/24 h).

### 4.4. Effect on Intracellular Amastigotes and Host Toxicity

The macrophages were collected from the peritoneal cavity of uninfected male Swiss mice (5–6 weeks) after the injection of 8 mL of RPMI medium. The peritoneal macrophages (3 × 10^5^ cells/well) were resuspended in RPMI and plated in 24-well plates (Nunc Inc.) and then infected with trypomastigotes (10 parasites/host cell). After 24 h of incubation, the cultures were washed to remove non-internalized parasites, and Pgd, PmTE and Efr were added (1.5 to 6 μg/mL) for 24 h at 37 °C. The results were expressed using the IC_50_/24 h concentration, which corresponded to the value that led to a 50% decrease in the infection index (percentage of infected host cells multiplied by the number of parasites per cell) [44].

Additionally, non-infected macrophages (5 × 10^4^ cells/well) were also treated with the three substances for 24 h at 37 °C for the toxicity analysis of host cells. After the treatment, 10 µL PrestoBlue (Invitrogen, Carlsbad, CA, USA) was added to the final concentration of 10% for 2 h at the same temperature. The measurements were performed at 560 and 590 nm, as recommended by the manufacturer at SpectraMax M3 fluorimeter (Molecular Devices, San Jose, CA, USA). The results were expressed as LC_50_/24 h, which corresponds to the concentration that leads to damage of 50% of the host cells. Finally, the relationship between the trypanocidal effect and host toxicity was expressed as the selectivity index (SI) calculated by LC_50_/24 h for uninfected macrophages per IC_50_/24 h for intracellular amastigotes.

### 4.5. Ultrastructural Analysis

Epimastigotes (5 × 10^6^ parasites/mL) were treated in LIT medium at 28 °C for 24 h with Pgd (42 and 85 µg/mL), PmTE (20 and 40 µg/mL), and Efr (25 and 50 µg/mL), never exceeding their respective IC_50_/24 h dose. The parasites were fixed with 2.5% glutaraldehyde in 0.1 M sodium cacodylate buffer (pH 7.2) for 40 min at room temperature and post-fixed with 1% OsO_4_, 0.8% potassium ferricyanide and 2.5 mM CaCl_2_ in the same buffer and temperature for 20 min. Then, the samples were dehydrated in an ascending acetone series, embedded in PolyBed 812 resin, cut into ultrathin sections and stained with uranyl acetate and lead citrate. The analysis of three independent biological replicates (at least 30 sections per experimental condition) was performed in a JEM1011 transmission electron microscope (JEOL, Tokyo, Japan) located in the Plataforma de Microscopia Eletrônica at Instituto Oswaldo Cruz (Fiocruz, Rio de Janeiro, Brazil).

### 4.6. Flow Cytometry Analysis

Epimastigotes (5 × 10^6^ parasites/mL) were treated with Pgd (21–85 µg/mL), PmTE (10–40 µg/mL), and Efr (12–50 µg/mL) for 24 h, and then the mitochondrial membrane potential (ΔΨm), ROS production and plasma membrane integrity were assessed. For ΔΨm analysis, the parasites were incubated with 32 ng/mL Rh123 (Sigma-Aldrich, St. Louis, MO, USA) for 30 min at 28 °C, using 10 µM carbonyl cyanide 4-(trifluoromethoxy)phenylhydrazone (FCCP) (Sigma-Aldrich) to dissipate ΔΨm as a control. Rh123 labelling was quantified using an IV, which was calculated by the equation (MTMC)/MC, where MT is the median of fluorescence for treated parasites and MC is the median of fluorescence of the control parasites, and negative IV values correspond to mitochondrial depolarization [39]. The incubation with FCCP was employed to normalize the data, minimizing the unspecific labelling of this marker. For the evaluation of ROS production, epimastigotes were labelled with 10 µM DHE (Molecular Probes, Carlsbad, USA) for 30 min at 28 °C, and the incubation with 10 µM menadione (Sigma-Aldrich), used as a positive control for the generation. For the analysis of plasma membrane permeabilization, the parasites were incubated with 10 µg/mL PI (Sigma-Aldrich) for 20 min, and 0.1% saponin was added as a control of permeabilization. All assays were performed in a FACSCalibur flow cytometer (Becton Dickinson, Franklin Lakes, NJ, USA) equipped with Cell Quest software (Joseph Trotter, Scripps Research Institute, La Jolla, CA, USA). A total of 10,000 events were acquired in the region previously established as that of the parasites.

### 4.7. Statistical Analysis

Statistical analyses were performed using the non-parametric Mann-Whitney test in the IBM SPSS Statistics 22.0 software (IBM Corporation, Armonk, NY, USA), with the threshold for significanceset at *p* ≤ 0.05. In all experiments, the pairwise comparisonswerecontrolversustreated parasites. Comparisons among the different doses andcompounds were not performed.

## 5. Conclusions

In conclusion, isolated sesquiterpenoids Pgd and Efr were the most active substances in all *T. cruzi* stages (bloodstream trypomastigotes, epimastigotes and intracellular amastigotes) and presented low host toxicity. Electron microscopy analysis demonstrated reservosome disruption, cytosolic vacuolization, autophagic phenotype and mitochondrial swelling as the most recurrent morphological effects. Pgd also led to important effects on the parasite mitochondrion, including organelle swelling and loss of its membrane potential, together with ROS generation and subsequent lysis of the protozoa. The description of anti-*T. cruzi* effects of these sesquiterpenoids can represent a useful step for the search for alternative drugs for Chagas disease. Finally, the search for natural products, especially those with potential biological activities in folk medicine, could be an excellent alternative for the discovery of novel anti-*T. cruzi* drugs.

## Figures and Tables

**Table 1:** Trypanocidal activity of total extracts and isolated sesquiterpenoids on different forms of *T. cruzi*.

Extract/Compound	IC_50_/24 h (μg/mL)			LC_50_/24 h (μg/mL)	SI ^*c*^
	Trypomastigotes	Epimastigotes	Amastigotes ^*a,b*^		
*D. winteri* TE *^d^*	77.8 ± 2.3 *^e^*	174.9 ± 23.2	- *^f^*	-	-
Polygodial (Pgd)	11.5 ± 2.1	84.4 ± 9.4	1.0 *^f^* ± 0.1	10.1 ± 2.3	10.3
Cinnamolide	196.3 ± 24.6	>4000.0	-	-	-
Dendocarbin A	>800.0	>500.0	-	-	-
Isodrimeninol	50.5 ± 5.9	169.9 ± 19.6	-	-	-
*P. mitiqui* TE (PmTE)	5.6 ± 0.6	40.8 ± 5.3	1.0 ± 0.2	20.7 ± 0.6	21.5
Erioflorin acetate (Efr)	6.1 ± 1.6	55.5 ± 5.4	0.9 ± 0.1	24.9 ± 3.8	29.5
*M. boaria* TE	>2500.0	>1500.0	-	-	-
MB-16	>4000.0	>2500.0	-	-	-
MB-22	>1000.0	>4000.0	-	-	-

*^a^* Intracellular amastigotes in peritoneal macrophages; *^b^* IC_50_/24 h of intracellular amastigotes was based on the infection index (percentage of infected host cells multiplied by the number of parasites per cell); *^c^* SI = LC_50_/24 h per IC_50_/24 h for amastigotes; *^d^* TE = total extract; *^e^* Mean ± standard deviation of at least three independent experiments; *^f^* Not determined.

**Table 2:** Flow cytometry analysis of mitochondrial membrane potential in *T. cruzi* epimastigotes.

Compound	Dose	Median	IV ^*a*^
Control	-	72.6 ± 24.6 *^b^*	0.0
Pgd	85 μg/mL	12.8 ± 2.8 *	−0.83
	42 μg/mL	14.6 ± 5.4 *	−0.80
	21 μg/mL	55.9 ± 12.0	−0.23
Efr	50 μg/mL	4.1 ± 0.6 *	−0.90
	25 μg/mL	8.4 ± 2.9 *	−0.88
	12 μg/mL	58.6 ± 22.8	−0.60
PmTE	40 μg/mL	7.4 ± 5.8 *	−0.94
	20 μg/mL	27.9 ± 5.2 *	−0.93
	10 μg/mL	29.0 ± 20.4 *	−0.56

*^a^* IV = (MT−MC)/MC, where MT corresponds to the median fluorescence for treated parasites, and MC corresponds to that of control parasites; *^b^* Mean ± standard deviation of three independent experiments; *Asterisks indicate significant differences in relation to the untreated control group (*p* = 0.05) by Mann-Whitney test.

**Table 3:** Flow cytometry analysis of ROS production in *T. cruzi* epimastigotes.

	Dose	Median	IV ^*a*^
Control	-	10.8 ± 4.0 *^b^*	0.0
Menadione	10 μM	44.1 ± 4.9 *	4.1
Pgd	85 μg/mL	66.1 ± 6.6 *	6.1
	42 μg/mL	63.4 ± 6.4 *	5.9
	21 μg/mL	66.8 ± 5.0 *	6.2
Efr	50 μg/mL	38.8 ± 5.5 *	4.7
	25 μg/mL	33.4 ± 3.5 *	3.9
	12 μg/mL	32.9 ± 4.1 *	3.1
PmTE	40 μg/mL	50.6 ± 5.3 *	3.6
	20 μg/mL	41.6 ± 12.3 *	3.1
	10 μg/mL	33.7 ± 6.8 *	3.1

*^a^* IV = MT /MC, where MT corresponds to the median fluorescence for treated parasites, and MC corresponds to that of control parasites; *^b^* Mean ± standard deviation of four independent experiments; * Asterisks indicate significant differences in relation to the untreated control group (*p* ≤ 0.02) by Mann-Whitney test.

## Supplementary Materials

Supplementary data associated with this article can be found in the online version.
