# Supplementary material for: TrypanocidalActivity of Natural Sesquiterpenoids Involves Mitochondrial Dysfunction, ROS Production and Autophagic Phenotype in Trypanosoma cruzi
Source: Molecules. 2018 Oct 28;23(11):2800. doi: 10.3390/molecules23112800 (PMC6278339; doi:10.3390/molecules23112800)
Supplement: Supplementary file 1 [file molecules-23-02800-s001.pdf]

Trypanocidal activity of natural sesquiterpenoids involves mitochondrial disfunction, ROS production and autophagic phenotype in *Trypanosoma cruzi*

Ana Cristina S. Bombaça[1], Daniela Von Dossow[2], Juliana M. C. Barbosa[1], Cristian Paz[2,*], Viviana Burgos[2], Rubem F. S. Menna-Barreto[1,*]

[1]Laboratório de Biologia Celular, Instituto Oswaldo Cruz, Fundação Oswaldo Cruz, Rio de Janeiro, Brazil;
anabombaca@gmail.com (A.C.S.B.); julianabmunirio@gmail.com (J.M.C.B.)
[2]Departamento de Ciencias Químicas y Recursos Naturales, Universidad de La Frontera, Temuco, Chile;
d.vondossow01@ufromail.cl (D.V.D); viviana.burgos@ufrontera.cl (V.B.)

*Corresponding authors at: cristian.paz@ufrontera.cl - Francisco Salazar 01145, Temuco, Chile - Tel: +56(45)2325424 (C.P.); rubemsadok@gmail.com - Av. Brasil, 4365, Rio de Janeiro, Brazil - Tel: +55(21)25621393 (R.F.S.M.B)

Supplementary File 1

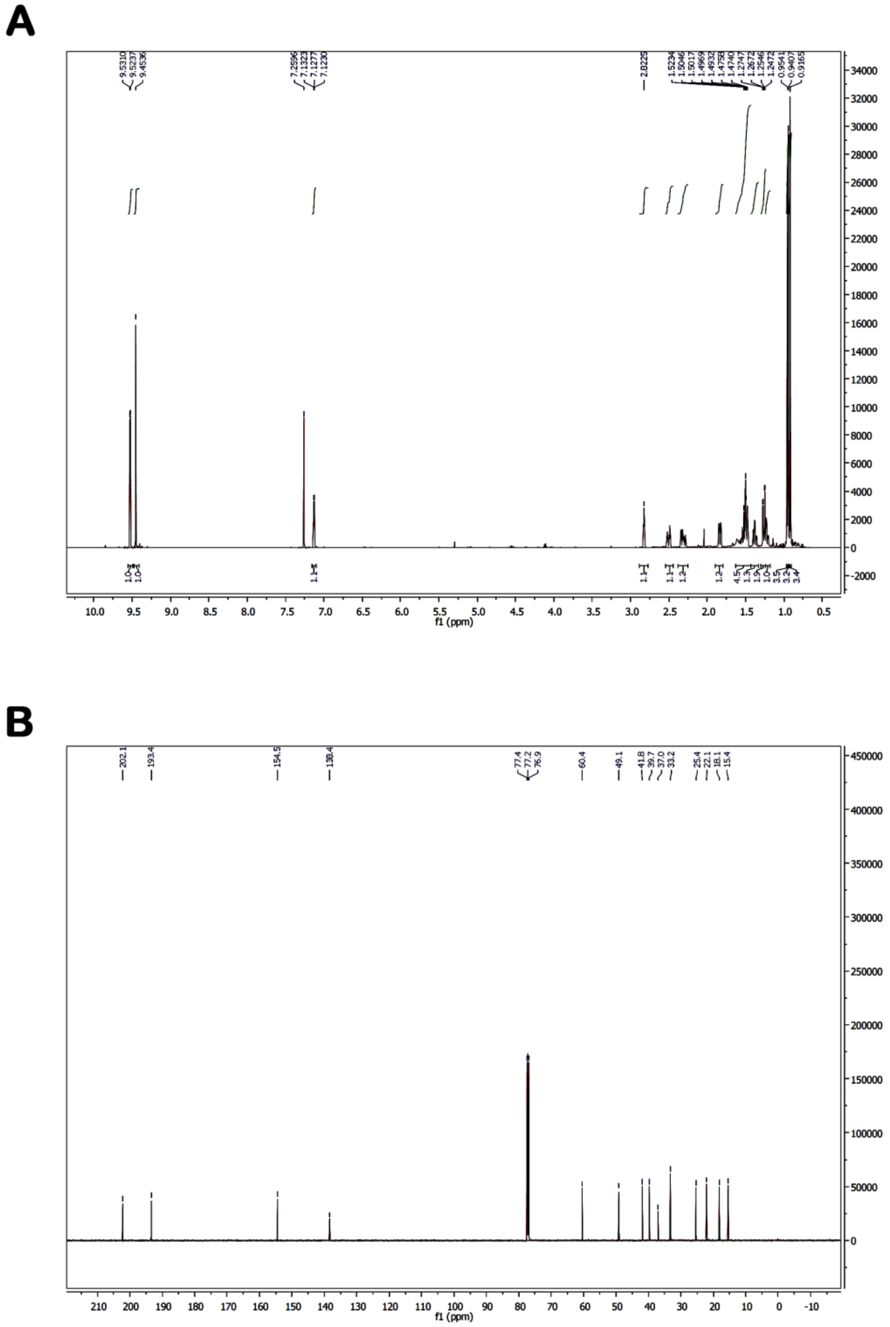

Figure S1. Pgd NMR spectra. A. ^{1}H NMR spectra (at 600 MHz in $CDCl_3$). B. ^{13}C NMR spectra (at 150 MHz in $CDCl_3$).

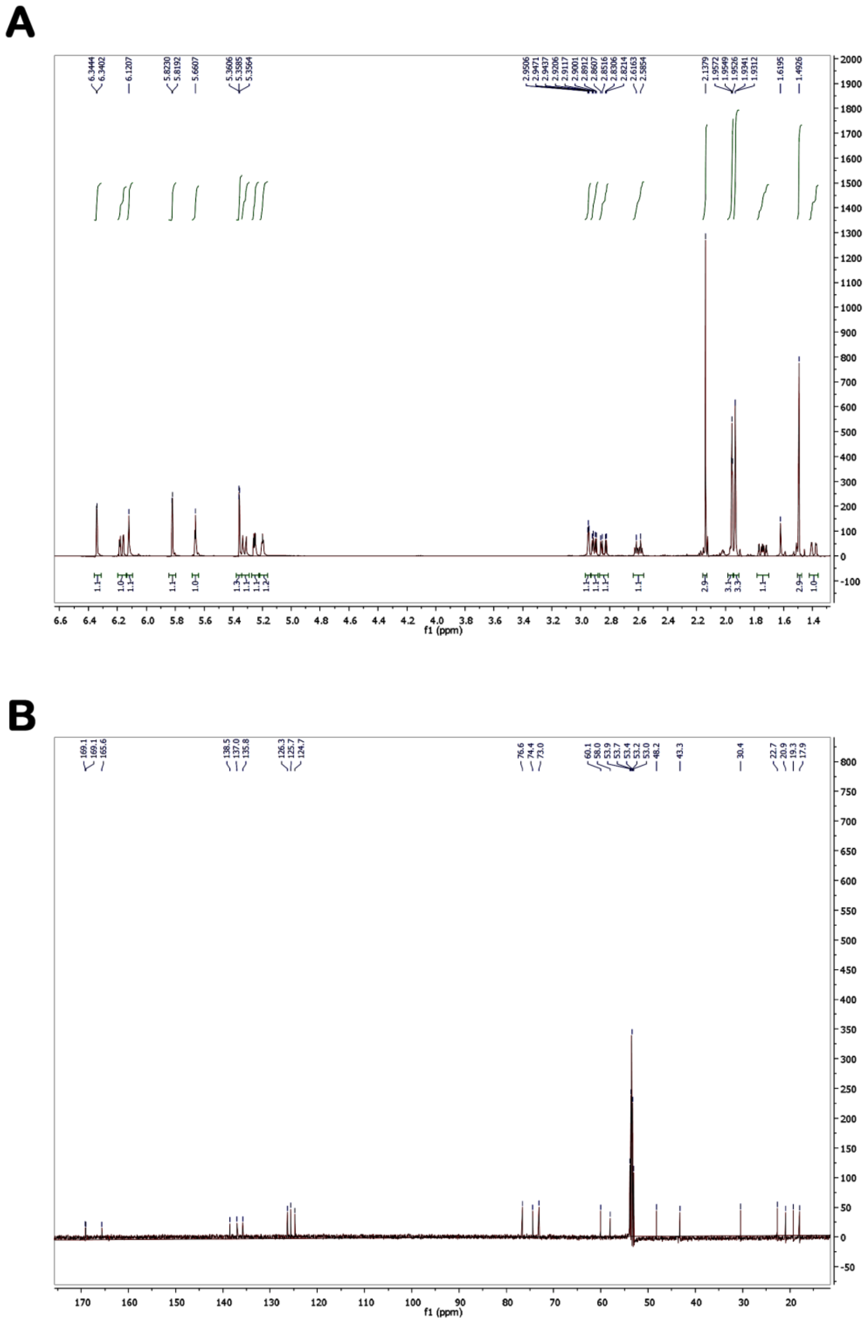

Figure S2. Efr NMR spectra. A. ^{1}H NMR spectra (at 600 MHz in DMSO-d_6). B. ^{13}C NMR spectra (at 150 MHz in DMSO-d_6).

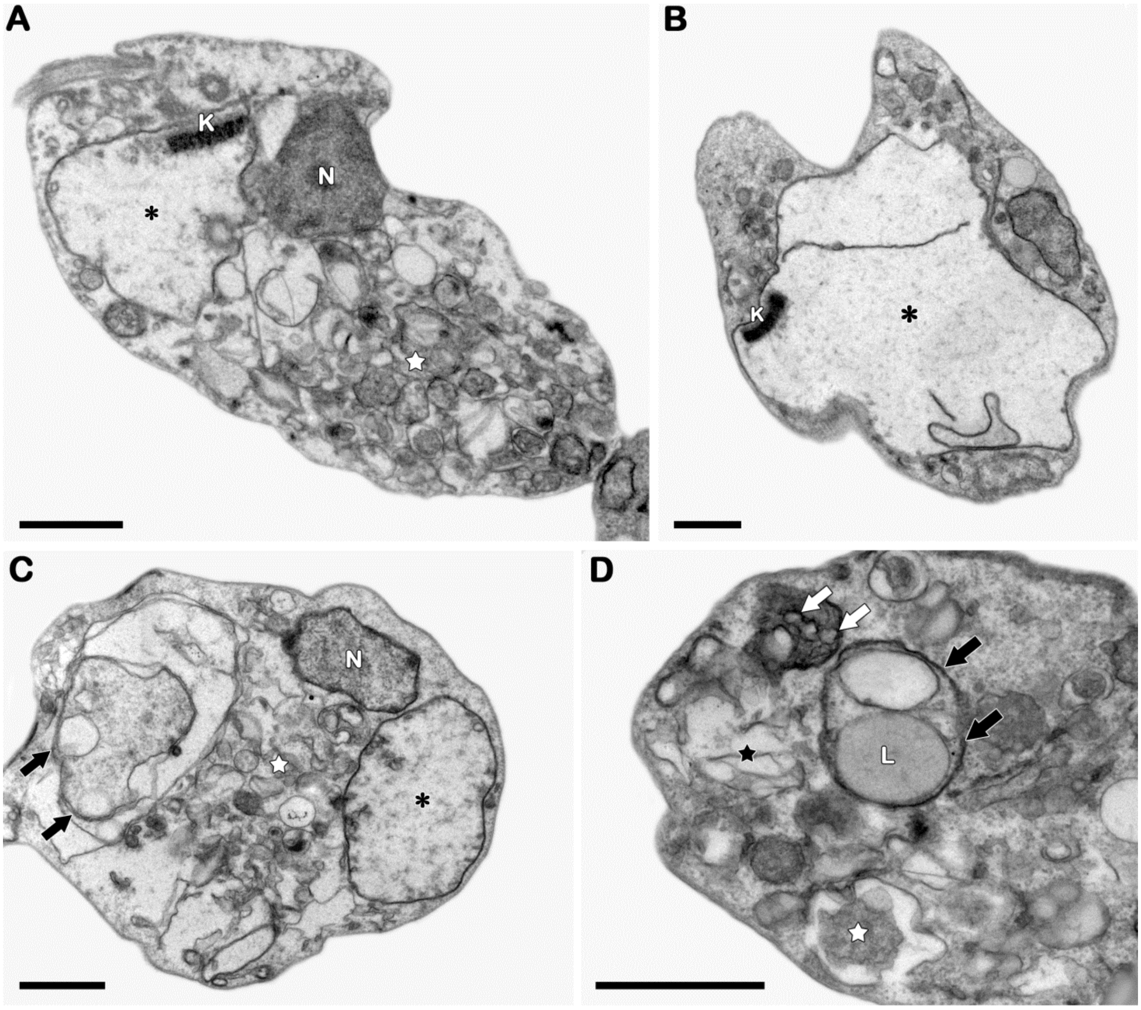

Figure S3. Transmission electron microscopy analysis of *T. cruzi* epimastigotes treated with 85 µg/mL drimane sesquiterpenoid Pgd. (A-D) As in parasites treated with a lower concentration, the IC_{50}/24h dose induced a recurrent mitochondrial swelling (black asterisks), with abnormal cristae morphology (white arrows), and disorganization of the reservosomes (black star). The treatment with Pgd also led to an autophagic phenotype with a formation of a great number of autophagosomes (white stars) as well as the presence of endoplasmic reticulum profiles (black arrows) surrounding subcellular structures. N: nucleus, K: kinetoplast, L: lipid droplet. Bars in A, B and D = 1 µm; Bar in C = 0.5 µm.

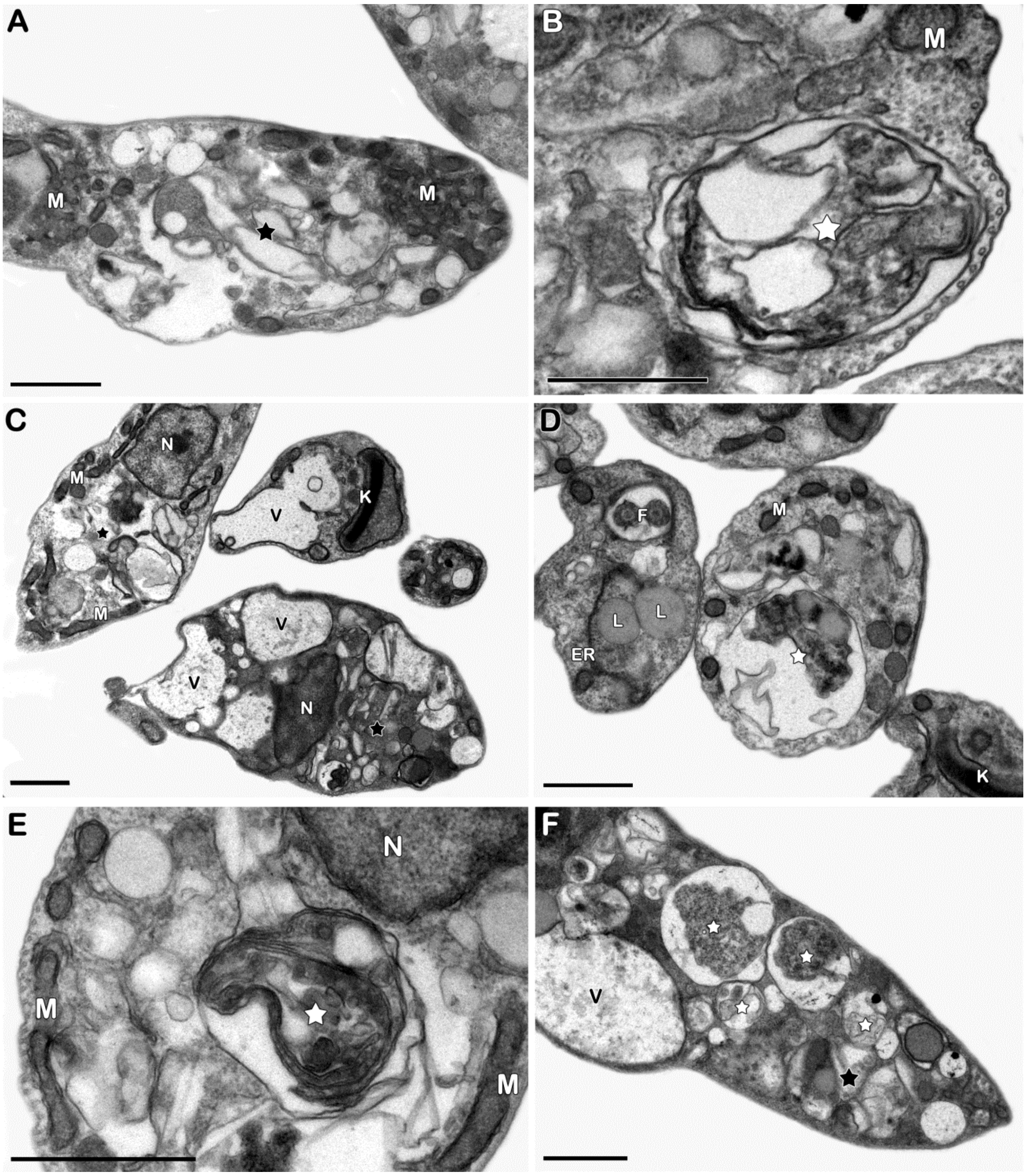

Figure S4. Transmission electron microscopy analysis of *T. cruzi* epimastigotes treated with PmTE. The treatment with (A,B) 20 and (C-F) 40 µg/mL of this total extract led to an intense disorganization of reservosomes (black stars), autophagic phenotype clearly demonstrated by the increase in the number of autophagosomes (white stars) with distinct stages of cargo degradation, as well as the important cytosolic vacuolization (V). N: nucleus, M: mitochondrion, K: kinetoplast, ER: endoplasmic reticulum, L: lipid droplets. Bars in A and C-F = 1 µm; Bar in B = 0.5 µm.
